# Somatic Comorbidity of Anxious and Depressed Miners

**DOI:** 10.1192/j.eurpsy.2022.1174

**Published:** 2022-09-01

**Authors:** N. Becarevic, R. Softic, M. Becarevic

**Affiliations:** 1 University Clinical Center Tuzla, Of Psychiatry, Tuzla, Bosnia and Herzegovina; 2 Health Care Center Banovici, Family Medicine, Banovici, Bosnia and Herzegovina

**Keywords:** Antidepressants, Depression, miners, comorbidity

## Abstract

**Introduction:**

Depression is the leading cause of disability worldwide and is a major contributor to the overall global burden of disease. The prevalence of depression is rising and it often co-occurs with other physical diseases.

**Objectives:**

The research aims to determine the comorbidity of depression and anxiety disorders with chronic physical diseases among employees of the „Brown coal mine Banovici“.

**Methods:**

We conducted a retrospective study that included 117 employees from the disease registry who are being under the treatment of depression and anxiety disorder. We collected data from medical records of patients about sex, age, marital status, smoking status, physical diseases, types of antidepressants, and the other drugs they use.

**Results:**

The study showed that there are 117 employees of the „Brown coal mine Banovici“ who are under treatment of depression and anxiety-depressive disorder. 22 (18,8%) of them are females and 95 (81,2%) males in an average life span of 48,3 years. The most commonly used antidepressant is Escitalopram. 62 (53%) out of 117 patients with depression have comorbidity with diseases of the circulatory system, 24 (20,51%) have comorbidity with diseases of the musculoskeletal system and connective tissue, 16 (13,68%) have comorbidity with endocrine, nutritional and metabolic diseases. 25 (21,37%) patients are not suffering from any other chronic physical disease. The most commonly used drugs besides antidepressants are antihypertensives.

**Conclusions:**

The comorbidity rate of depression and anxiety disorders with cardiovascular diseases among employees of the „Brown coal mine Banovici“ is higher than with all other chronic physical diseases.

**Disclosure:**

No significant relationships.

